# Attitudes and Beliefs about Placebo Surgery among Orthopedic Shoulder Surgeons in the United Kingdom

**DOI:** 10.1371/journal.pone.0091699

**Published:** 2014-03-14

**Authors:** Karolina Wartolowska, David J. Beard, Andrew J. Carr

**Affiliations:** Botnar Institute of Musculoskeletal Sciences, Nuffield Department of Orthopedics, Rheumatology and Musculoskeletal Sciences, University of Oxford, Oxford, United Kingdom; University of Louisville, United States of America

## Abstract

**Objectives:**

To survey surgeons on their beliefs and attitudes towards the use of placebo in surgery.

**Methods:**

British orthopedic shoulder surgeons, attending a national conference in the United Kingdom, were asked to complete a self-report online questionnaire about their beliefs and attitudes towards the use of placebo related to surgical intervention. The survey included questions about ethical issues, the mechanism of placebo effects, and any concerns regarding its use.

**Results:**

100 surgeons who participated in the survey believed that placebo surgery is ethically acceptable (96%), especially as a part of a clinical trial (46%). Respondents thought that a placebo effect in surgery is real i.e. has a scientific basis (92%), that placebo can be therapeutically beneficial (77%), and that it involves psychological mechanisms (96%). Over half of the respondents (58%) have used a surgical procedure with a significant placebo component at least once in their professional career. Their main concern about placebo use in surgery was that it might involve an element of deception.

**Conclusions and Implications:**

Surgeons generally agreed that a placebo component to surgical intervention might exist. They also supported placebo use in clinical trials and considered it ethical, providing it does not involve deception of patients. More studies are needed, particularly among other surgical specialties and with larger numbers of participants, to better understand the use of placebo in surgery.

## Introduction

The placebo effect is a behavioral and clinical response to the meaning of treatment. The term refers to the improvement related to a placebo, i.e., an inactive substance or a procedure without the therapeutic element. An actual intervention, pill, or injection is not necessary and even a positive consultation, a definite diagnosis, or a diagnostic test may have placebo effects [1]–[4]. The placebo effect is influenced by the previous experiences as well as the beliefs and expectations about the treatment [5]–[7].

Placebo is frequently used in pharmacological randomized clinical trials but its role in surgical trials is less widely accepted [8]–[10]. As surgery is now often performed to improve quality of life, as opposed to the preservation of life, the outcomes measured are more subjective and often depend on patients' and assessors' perception of pain, function, or quality of life. These subjective outcomes are prone to bias in an open-label design [11]. A placebo effect, as well as its negative counterpart, i.e., nocebo effect, has to be considered while interpreting results of trials with subjective outcomes, especially if the group with active intervention is compared with patients without surgery, e.g., the waiting list. Therefore, to distinguish the results of surgery from the non-specific effects of undergoing treatment, any comparative study must include 1) the intervention under investigation and 2) a placebo, i.e., a procedure that imitates the surgical intervention and is indistinguishable from it but does not involve the specific therapeutic component.

Surgical placebo raises more ethical concerns than placebo pills [12]–[14] because it is potentially associated with more risk as it inherently involves tissue incision and either anaesthesia or sedation. Moreover, in order to design a randomized trial one has to assume that the null hypothesis is true. However, the notion that any given surgical procedure is ineffective, particularly a surgical procedure they have recommended and performed, can create apprehension for surgeons, as they are personally involved in the treatment.

Although, placebo use has been the subject of many ethical debates, there have been very few studies investigating the attitudes of doctors towards placebo, the conditions under which they find it acceptable, and the constraints in its use [15], [16]. According to a systematic review of the existing studies, between 17 and 80% of doctors have used placebo in their practice [16]. A recent study reported that 97% of general practitioners (GPs) in the United Kingdom (UK) have used some form of placebo such as non-essential examinations or conventional medicine for which there was no evidence-based confirmation of efficacy [15]. There are only a few studies, in which surgeons have been asked about their attitudes towards placebo [17], [18].

The aim of this study was to formally survey a large group of British shoulder orthopedic surgeons about their beliefs and attitudes to placebo in the context of surgery. The surgeons were approached during a national conference at which a placebo-controlled trial was presented; an event that could have potentially raised awareness of surgical placebo and prompted discussions on this subject.

## Materials and Methods

### Participants

Surgical members of the British Elbow and Shoulder Society (BESS) participating in a national conference were invited via email to participate in an online survey. The data collection was open between 17th and 27th June 2013. This group of surgeons was chosen because of their awareness of a multi-center placebo-controlled trial for shoulder pain that was being carried out in the UK. Currently, 14 orthopedic centers in the UK are involved in the trial of the efficacy of arthroscopic decompression for shoulder pain (CSAW, http://clinicaltrials.gov/show/NCT01623011). As the participants entered their responses directly into an online survey and the data were analyzed anonymously, it is not known whether the surgeons involved in this trial participated in the survey. This survey was performed in relation to the CSAW trial in which all the authors are involved. This study was reviewed by the Institutional Board at the Nuffield Department of Orthopaedics, Rheumatology and Musculoskeletal Sciences, which decided it did not require an ethics number as it is an anonymyzed survey of healthcare professionals.

### Survey instrument

A questionnaire used in this study was derived from previously published surveys to make the results comparable with earlier studies [15], [16], [19]–[21]. Participants were asked about their age, gender, and number of years since their professional accreditation as well as the location and size of their practice. As it has been suggested that the confusion surrounding the ‘placebo’ concept may be an obstacle to investigating placebo use [15], the placebo-specific part of the questionnaire started with a closed question on the definition of placebo. Surgeons were asked whether they agreed with a provided definition of surgical placebo as “an intervention where patients undergo a surgical procedure that has the appearance of a therapeutic intervention, but during which the essential therapeutic manoeuvre is omitted” [22]. Space was provided to allow further comments related to the definition. In this survey participants were only asked about the placebo effects. Two further closed questions asked whether the respondents believed that the placebo effect is real, i.e., there is a scientific basis, and whether it has a therapeutic benefit. The next three questions were related to situations in which the surgeons would consider a placebo, the concerns related to placebo, and possible mechanisms of placebo effects. These questions had several possible answers and respondents had an option to choose more than one answer. The participants were also asked how often they used operations that they believe may have a significant placebo component. The questionnaire ended with a direct question about the participant's ethical stance and overall position concerning the use of placebo.

### Data analysis

Participants entered their responses directly into an online survey. [The website is available at: http://www.surveymonkey.net/; last accessed on 27^th^ June 2013]. The data were summarized using percentages and cross-tabulations to describe the responses to each question.

## Results

### Respondent characteristics

Of the 195 surgeons who were invited to participate in the survey 100 (51.3%) replied. As the data were anonymized it is not possible to establish whether the consultants involved in the placebo-controlled trial participated in the survey.

**Figure 1:**
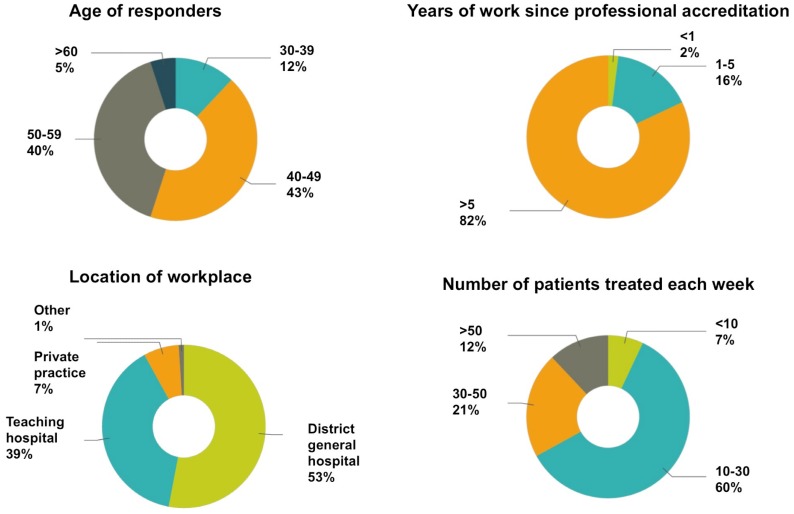
Respondent characteristics.

The respondents were mostly male (94%), consultants (96%), and aged between 40 and 60 years (83%). 82% of respondents have been professionally accredited for more than five years. The majority of the participants worked at a district general hospital (53%) or a teaching hospital (39%), and treated 10 to 30 patients per week (60%) (Figure 1).

### Definition of placebo

The majority of respondents (90%) agreed with the provided definition of placebo. Thirteen surgeons gave additional comments; including three on the semantics (i.e., whether it should be called “a placebo surgery” or “sham”), seven on the fact that a placebo does not necessarily have to involve a surgical procedure, and three commented on the fact that the “essential manoeuvre” is often unknown or difficult to determine. Ten participants who disagreed with the definition did not comment on their reasons.

### Beliefs about placebo effects

Most of the respondents believed that the placebo effect is real (92%) and that it has therapeutic benefits (77%) and these two factors were associated (Fisher's exact test significant at p = 0.02) (see also Table 1).

**Table 1:** Interactions between beliefs about placebo, the ethical stance and the clinical practice.

		Do you believe that placebo has a therapeutic benefit?	
		Yes	No
**Do you believe that the placebo effect is real?**	**Yes**	**N = 75**	**N = 17**
		**Frequency of use:**	**Frequency of use:**
		>1 per month N = 13	>1 per month N = 0
		>1 per year N = 20	>1 per year N = 3
		<1 per year N = 18	<1 per year N = 2
		Never N = 24	Never N = 12
		**Position on placebo use:**	**Position on placebo use:**
		Always prohibited N = 2	Always prohibited N = 1
		Permitted* if supported by research N = 25	Permitted* if supported by research N = 5
		Permitted* if supported by experience N = 16	Permitted* if supported by experience N = 1
		Permitted only in clinical trials N = 32	Permitted only in clinical trials N = 10
	**No**	N** = **2	N** = 6**
		**Frequency of use:**	**Frequency of use:**
		>1 per month N = 0	>1 per month N = 0
		>1 per year N = 1	>1 per year N = 0
		<1 per year N = 1	<1 per year N = 0
		Never N = 0	Never N = 6
		**Position on placebo use:**	**Position on placebo use:**
		Always prohibited N = 0	Always prohibited N = 1
		Permitted* if supported by research N = 1	Permitted* if supported by research N = 2
		Permitted* if supported by experience N = 0	Permitted* if supported by experience N = 0
		Permitted only in clinical trials N = 1	Permitted only in clinical trials N = 3

### Indications for placebo use

The three most common situations in which the respondents would consider use of placebo were 1) to distinguish between organic and non-organic symptoms (34%), 2) when all other therapies have been exhausted (26%), and 3) as a treatment for non-specific symptoms (20%) (Table 2). 34% of respondents would not use placebo in any of the given situations, including 21 surgeons who reported that they had never used a procedure with a placebo component as well as the three surgeons who reported using such procedures often.

**Table 2:** Opinions regarding placebo mechanisms, possible use and concerns regarding its use.

**In which of the following situations, have you considered placebo? (more then one option allowed)**	
None of the above	34%
As a diagnostic tool	34%
When all other therapies have been exhausted	26%
As a treatment for a nonspecific symptom	20%
Instead of surgery, when using surgery was not justified	16%
As a supplement to surgery	15%
To calm a patient or to mollify a complaining patient	15%
To control pain	12%
To maintain a good relationship with a patient	9%
**What are your concerns regarding the use of placebo in surgery?**	
**(more than one option allowed)**	
It involves deception	62%
It endangers the patient-surgeon trust	48%
Because of legal problems	43%
Because of possible side effects	34%
It is ineffective	15%
None of the above	2%
**Scientific data suggest that some patients benefit from the placebo effect.**	
**In your opinion, what is the mechanism behind this phenomenon?**	
**(more than one option allowed)**	
Psychological	95%
Unexplained factors	46%
The natural course of the illness	41%
Conditioning	24%
Physiological	23%
Positive energies	7%
Other	3%

### Concerns regarding placebo use

The surgeons were mainly concerned about the element of deception associated with the use of placebo (62%). They were also apprehensive about the risk to the patient-surgeon trust (48%), potential legal problems (43%), or possible side effect of the placebo intervention (34%). It is worth noting that 15% of respondents believed that placebo is ineffective but only four of them thought that it should be also prohibited (Table 2).

### Mechanisms of placebo

The majority of the participants (95%) believed that the placebo effect is a result of psychological mechanisms. Only 24% thought that conditioning may be important, and 23% thought placebo effect has a physiological basis. Almost half of the surgeons replied that the placebo effect is a result of unexplained factors (46%) or a result of the natural course of the illness (41%) (Table 2).

### Frequency of placebo use in surgery

**Figure 2:**
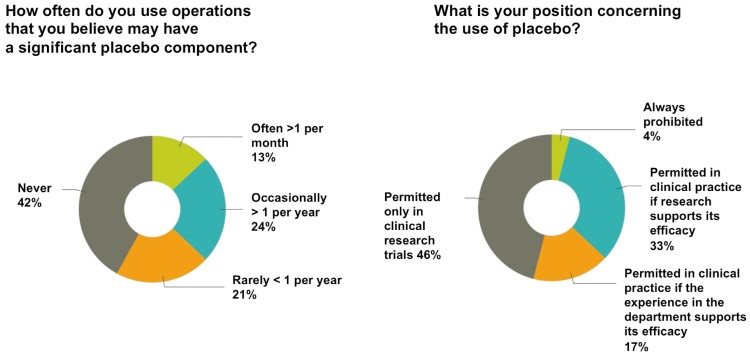
Frequency of placebo use and ethical stance of respondents.

Less than half of the surgeons (42%) responded that they had never used any surgical procedure that may have a significant placebo component. About 13% thought that placebo effects were present in their practice for more than one procedure per month. Others reported that they used such a procedure more than once a year (24%) or less than once a year (21%)(Figure 2). Surgeons who used procedures with a placebo component often or occasionally were those who also reported that the placebo effect was real and it had a therapeutic benefit (Table 1).

### Ethical attitudes

Almost half of the respondents (46%) believed that placebo has a role only in clinical research trials. Some answered that placebo is permitted in clinical practice if research supports its efficacy (33%) or if the experience in the department supports its efficacy (17%)(Figure 2). Only 4% of surgeons responded that placebo use should always be prohibited; however, half of these still believed that the placebo effect has a therapeutic benefit (Table 1).

## Discussion

Surgeons are not generally opposed to placebo. The majority of surgeons who responded to the survey found surgical placebo ethically acceptable and over half of the respondents reported that they had used a procedure with a significant placebo component at least once in their professional career. Most of the surgeons believed that placebo has a real and therapeutic effect. Interestingly, only those who believed in actual positive effect of placebo would also report frequent use of procedures with placebo element; however, others who shared these beliefs reported that they have never used such a procedure. Moreover, some of the respondents, who answered that the placebo effect is real and therapeutic also believed that its use should be prohibited. The main reason why surgeons would consider using placebo was to distinguish organic from non-organic symptoms and their main concern was the element of deception associated with the use of placebo.

The response in this survey was comparable to other studies (46–57%) [15], [20], [23]. The sample size was smaller than in the recent survey among the GPs [15] but it was larger in comparison to many earlier studies [16]. There was a potential response bias as the use of placebo interventions in clinical practice is ethically, professionally and legally controversial [16]; therefore, it is possible the surgeons either did not participate in the survey or understated their use of placebo, as has been suggested in other studies [15], [19]. As the present study was a retrospective self-report questionnaire, recall might also be a problem. Although, it has been argued by Nitzan and Lichtenberg [19] that the fact of using placebo was probably remembered. The shoulder surgery community in the United Kingdom was aware that a placebo-controlled surgical trial of shoulder surgery (CSAW) is recruiting. All 100 respondents attended the annual national meeting just prior to the survey, including the 14 investigators in the trial. The results were anonymized preventing a comparison between trialists and non-trialists. The study sample was representative for the surgeons in the UK. According to Royal College of Surgeons census from 2011, 95.8% of consultants in orthopedic surgery in the UK are male and about 80% of are in their 40 s and 50 s [24]. The predominance of male respondents might have biased the responses [15] although some studies have reported lack of interactions between gender and attitude to placebo [19].

According to earlier surveys among healthcare professionals, only a small number of doctors (2–12%) object to the use of placebo and believe that it should be prohibited [20], [21], [25], [26] [16]. In general, they believe that placebo is permissible in certain circumstances, especially if there is some evidence of its efficacy either from research or earlier experience. Moreover, the majority of respondents admits using placebo but very few are “frequent users” [15], [19]. According to a systematic review of surveys and qualitative studies among health care professionals “pure” placebos, such as saline injections, were used at least once in their career by between 17 and 80% of doctors [16]. “Impure” placebos, such as antibiotics for viral infections or non-essential examinations, were used even more frequently; with probably up to 99% percent of doctors using some form of placebo during their professional career. For example, in a recent study, up 97% of GPs in the UK have used some form of placebo during their career [15].

In the context of orthopedic surgery, there is no equivalent of an inert sugar pill because a placebo procedure has to be active and involve at least a skin incision and analgesia in order to imitate the surgical treatment. The CSAW trial as well as an earlier study by Moseley and colleagues [27] have chosen an arthroscopy as a placebo because it resembles decompression or debridement procedure but does not involve the surgical element that is believed to be therapeutic; however, it may still add some diagnostic value.

The frequency of placebo use reported by surgeons in this survey was lower than the frequency reported by primary care practitioners in earlier studies [15], [16]. This may be explained by the culture among surgeons with their direct involvement in the treatment, which, unlike pharmacological treatment, is a physical and skill-based intervention [28]. Interestingly, Shapiro observed that surgeons believe that they use placebos or non-specific treatment less often compared with other specialties [17], although the believe that surgery may have a placebo element [29].

It is important that surgeons are aware of the fact that the placebo effect may be a component of treatment, diagnostic tests or even consultations and that those effects cannot be ignored during any assessment of efficacy of any therapeutic procedure. The results of this survey suggest that surgeons do not fully understand the mechanisms involved in the phenomenon of placebo response. Most surgeons were aware that the placebo effect is a result of psychological phenomena but not all of them knew about the importance of conditioning. Moreover, about two in five believed that placebo effect is related to some unknown processes or they did not distinguish between placebo effects and non-specific changes related, for example, to the natural course of the disease. Also one in three surgeons thought that placebo response would be present only if the complaint is non-organic.

According to a systematic review of earlier surveys, between 16 and 50% of healthcare professionals believe that placebo is generally effective but the motivations for the use of placebo are often complex [16]. In hospital settings, it is often used to alleviate pain and anxiety [16]. Among GPs in the UK the main reason for prescribing placebo is to induce possible psychological effects (55%), to calm the patient (33%), because patient requested therapy (32%), and to treat non-specific complaints (31%) [15]. Between 4 and 75% of healthcare professionals use placebo for diagnostic purposes, this is to determine whether the reported symptoms are real [16]. In a report on feasibility of placebo-controlled surgical trials 51% of surgeons were supportive of surgical placebo as they wanted to know why treatment is not always effective and why patients keep coming back [18]. In this survey, most surgeons replied that they used placebo for diagnostic purposes, supposedly believing that a non-organic complaint would improve after placebo whereas an organic cause would not. Others would use placebo when there was no effective treatment available.

In this study, surgeons were mostly worried about deception. This was also the main concern for majority of doctors in earlier studies [15], [16]. Interestingly, patients are also not opposed to the idea of use of placebo surgery and to participation in a placebo-controlled surgical trial as long as they are sufficiently informed [18], [30], [31]. Avoiding deception and explaining the specifics of a placebo-controlled study is possible. For example, the patients who took part in the placebo controlled trials on dopaminergic cells transplants for Parkinson's disease understood the design and the rationale for the study, the need for a placebo arm as well as the differences between the arms [32].

While interpreting this study it is important to keep in mind that the members of the BESS were approached during a conference at which a placebo-controlled RCT was presented and that all 14 surgeons involved in this trial potentially participated in the survey. Therefore, the results of this study may be representative only for a sub-group of surgeons in one country. This could have introduced bias although it cannot be assumed that if surgeons took part in a placebo-controlled trial they fully approve of the use of placebo. It is important to remember that these circumstances could have also raised the awareness of placebo in surgery and stimulated a discussion about placebo in surgery.

This survey demonstrated that surgeons are not generally opposed to placebo. However, there is a need for more studies on the use of placebo in surgery and surgical research, preferably investigating different surgical specialties and in more than one country. It is necessary to understand the preferences regarding treatment among surgeons as well as the interactions between their beliefs, practice and ethical stance.
